# Altruistic Punishment and Between-Group Competition: Evidence from *n*-person Prisoner’s Dilemmas

**DOI:** 10.1007/s12110-012-9136-x

**Published:** 2012-05-25

**Authors:** Susanne Rebers, Ruud Koopmans

**Affiliations:** 1 Faculty of Social Sciences, Vrije Universiteit, De Boelelaan 1081, 1081 HV Amsterdam, The Netherlands; 2 Wissenschaftszentrum Berlin für Sozialforschung, Research Unit Migration, Integration, Transnationalization, Reichpietschufer 50, 10785 Berlin, Germany

**Keywords:** Collective action, Evolution, Cultural group selection, Punishment

## Abstract

Collective action, or the large-scale cooperation in the pursuit of public goods, has been suggested to have evolved through cultural group selection. Previous research suggests that the costly punishment of group members who do not contribute to public goods plays an important role in the resolution of collective action dilemmas. If large-scale cooperation sustained by the punishment of defectors has evolved through the mechanism of cultural group selection, two implications regarding costly punishment follow: (1) that people are more willing to punish defecting group members in a situation of intergroup competition than in a single-group social dilemma game and (2) that levels of “perverse” punishment of cooperators are not affected by intergroup competition. We find confirmation for these hypotheses. However, we find that the effect of intergroup competition on the punishment of defectors is fully explained by the stronger conditionality of punishment on expected punishment levels in the competition condition.

Humans excel in large-scale cooperation for public goods, also known as collective action. Examples include participating in demonstrations, paying taxes, making donations to charities, or protecting natural resources. Public goods can only be obtained when several individuals cooperate, but once they are obtained it is not possible to exclude individuals from consuming them. That people often cooperate despite the fact that they could profit from the provisioning of public goods regardless of whether they contributed to their production has intrigued scholars, particularly sociologists and economists, for many years (e.g., Olson 1965). Recently, evolutionary scholars have also turned to this question (Boyd and Richerson 1982, 1985; Henrich 2004). Traditional explanations of the evolution of cooperation cannot explain this typical form of human cooperation (Henrich 2004). For instance, reciprocal altruism, commonly used to explain the evolution of cooperation among nonrelatives, cannot explain the evolution of collective action. Axelrod (1984) has shown with his famous computer tournaments that targeting cooperation toward those who cooperated, and targeting defections toward those who defected, is a successful strategy in the repeated, two-person prisoner’s dilemma. Reciprocal altruism, however, can only sustain cooperation in small groups (Boyd and Richerson 1988). In large groups, it is impossible to direct behavior to specific individuals depending on their previous behavior. Instead, in collective action one either cooperates with everybody or defects on everybody (Gil-White and Richerson 2003).

Punishment of uncooperating group members—often referred to as “altruistic punishment” (e.g., Barclay 2006; Boyd et al. 2003; Fehr and Gächter 2002; Fowler 2005)—has been suggested as an important mechanism for stabilizing collective action. If defectors must reckon with punishment, cooperating may become a rational, or at least a less irrational, thing to do (Richerson and Boyd 2005: chap. 6). Empirical data suggest that punishment is common in human societies (Boehm et al. 1993). Experimental research has shown that individuals are willing to punish group members (e.g., Fehr and Gächter 2000; Ostrom et al. 1992; Yamagishi 1986), even in one-shot interactions (e.g., Shinada and Yamagishi 2007). Cooperation levels in both ethnographic experiments and social dilemma games are higher when punishment is possible than in similar situations or games without this possibility (e.g., Bochet et al. 2006; Fehr and Gächter 2002; Heldt 2005b; Henrich et al. 2006; Masclet et al. 2003). Apart from the fear of being punished, people have also been shown to contribute more if the possibility of punishment exists because they expect others to contribute more (Shinada and Yamagishi 2007). The problem with the punishment solution to the collective action problem, however, is that it entails another collective action problem because punishing defecting group members entails costs to the punisher whereas the benefits of increased cooperation are enjoyed by the whole group, including those who did not participate in the costly punishment (Axelrod 1986; Marlowe et al. 2008; Yamagishi 1986).

A currently popular approach that offers a potential solution to these explanatory problems is cultural group selection. In short, the theory implies that when groups with different cultural traits, such as norms, values, or ideas, compete, groups with more group-beneficial cultural traits will replace the less-efficient groups. The proportion of individuals with group-beneficial traits will consequently increase in the global population (Boyd and Richerson 1982, 1985, 1990; Henrich and Boyd 2001).

As with other forms of collective action, punishment of defectors has been argued to have evolved through cultural group selection (e.g., Boyd et al. 2005; Richerson and Boyd 1999). Groups whose individuals were willing to punish those who did not contribute to the provisioning of public goods would have been better off in intergroup competition than other groups because cooperation can be sustained at a higher level when punishment is possible (Fehr and Fischbacher 2003; Gürerk et al. 2006). As a consequence, ideas, norms, and values leading to the punishment of defectors would spread (Boyd and Richerson 1982, 1985; Henrich and Boyd 2001). Importantly, some authors have argued that punishment of defecting group members can more easily evolve through cultural group selection than cooperation itself because the costs of punishment are strongly frequency-dependent: when defectors are rare, being a punisher is not very costly (Boyd et al. 2003; Henrich and Boyd 2001).

Three types of group selection that have been identified in the literature differ in the way in which cultural groups replace other groups (Henrich 2004; Van Veelen and Hopfensitz 2007). The first type is marked by differential population growth: some groups grow faster than others. This process is mathematically similar to the kin selection process (Lehmann et al. 2007; McElreath and Boyd 2007). The second type of cultural group selection is more subtle because the faster growth of some cultural groups is a result of their cultural traits being copied by neighboring groups more than other groups’ traits (e.g., Henrich 2004). In the third type of cultural group selection two groups directly compete for resources—for instance, through warfare. Owing to between-group differences, some cultural groups are more likely to survive this direct intergroup competition than other groups.

Importantly, only the third type of cultural group selection predicts differential behavior in situations with and without direct intergroup competition. In the first two types of cultural group selection, contributing to collective action can give groups with more group-beneficial cultural traits a selective advantage over other groups because they grow larger and form more daughter groups, or because their larger numbers or greater prosperity gives them a competitive edge once direct between-group conflict occurs. However, direct intergroup competition is unlikely to influence patterns of group-beneficial behavior as a consequence of these two processes because it is not required for these processes to take place. The theory of cultural group selection thus predicts that people will to some extent contribute to collective action when there is no direct between-group conflict. However, when a group is in direct competition with another group, as in the third type of group selection model, contributing to the group’s welfare is even more important for the group’s survival: “In times of peace they may compete for resources, but a war is survived together or not at all” (Van Veelen and Hopfensitz 2007). A model by Bowles (2009) showed that participation in warfare could indeed have had a significant influence on the evolution of the human capacity to participate in collective action.

The essential role of between-group competition in the third type of cultural group selection caused many authors to suggest that if this type of cultural group selection played a significant role in the evolution of collective action, a psychological predisposition to increase within-group cooperation for public goods in situations of between-group competition should have evolved (Burton-Chellew et al. 2010; Fehr and Fischbacher 2003; Puurtinen and Mappes 2009; Van Veelen and Hopfensitz 2007).

Experimental research has indeed shown that between-group competition increases within-group cooperation for a public good (Baron 2001; Baron et al. 2005; Bornstein and Ben-Yossef 1994; Burton-Chellew et al. 2010; Puurtinen and Mappes 2009; Reeve and Hölldobler 2007; West et al. 2006). There is however no experimental evidence on the influence of between-group competition on punishment behavior.

Given the emphasis on between-group competition as a key to the evolution of collective action, and the emphasis on its effect on cooperation in social dilemmas, it is striking that much of the experimental evidence on the role of punishment in social dilemmas derives from single-group experiments. If the human capacity to behave in an individually costly but group-beneficial way has evolved through cultural group selection, this should be reflected particularly in behavioral patterns in situations of group competition. In particular, two important corollaries follow from current theories of cultural group selection. First, if altruistic punishment has played a key role in the evolution of collective action through cultural group selection, punishment of defecting group members should be more prevalent in between-group competitive settings. As Fehr and Fischbacher (2003:790) have emphasized, this implication of the cultural group selection argument has not yet been empirically investigated. Second, previous research has shown that a small but non-negligible portion of participants punishes cooperating group members (Cinyabugama et al. 2006). Since this behavioral pattern is unlikely to increase within-group cooperation, and therefore cannot have been subject to cultural group selection, it is unlikely to increase with between-group competition. We thus expect that people display similar levels of punishment of cooperating group members in conditions with and without between-group competition.

We thus test two hypotheses in this paper:

1. In *n*-person prisoner’s dilemmas with a punishment option, people will be more inclined to punish defecting group members when there is between-group competition compared with single-group conditions.
2. In *n*-person prisoner’s dilemmas with a punishment option, there will be no significant difference in the tendency to punish cooperating group members between conditions with between-group competition and single-group conditions.

We investigate these hypotheses by comparing the behavior of participants in single-group and intergroup *n*-person prisoner’s dilemmas with a punishment option. For the intergroup *n*-person prisoner’s dilemmas, we utilize a slightly modified version of Bornstein and colleagues’ (Bornstein 1992; Bornstein and Ben-Yossef 1994) intergroup prisoner’s dilemma (hereafter: IPD). In the IPD, two groups of participants compete over a monetary bonus. The percentage of the bonus the members of a group receive depends on the number of cooperators in this group compared with the number of cooperators in the other group. Within each group, each member receives an equal share of the bonus, independent of whether or not this individual contributed to it. Further details on the distribution of money dependent on contribution decisions in both groups can be found in the methods section of this paper.

As did Bornstein and Ben-Yossef (1994), we implemented two versions of the single-group *n*-person prisoner’s dilemma game (PD): a “low payoff” and a “high payoff” version. In comparisons of participants’ behavior in the IPD and PD, having high and low payoff versions of the PD allows us to control for the effect of the absolute size of payoffs that can be made in the IPD. In all conditions, participants were given the opportunity to spend a portion of the earnings they made in the experimental game on punishing group members.

We opted for a two-stage design: each participant made one contribution decision in the IPD or PD, and then in the following round participants were allowed to punish group members. Since we were interested in determining whether participants had a stronger innate predisposition or preference to punish defectors in situations of between-group competition, we wanted to look at participants’ “intuitive” response to the PD or IPD. When participants play a social dilemma game for multiple rounds in the same group, punishment might be influenced by such strategic considerations as future gains, which were not the scope of this paper. A consequence of our decision is that this specific experiment cannot examine any difference in the influence of punishment on cooperation levels between the PD and the IPD since the positive influence of punishment on cooperation has been shown to be especially prevalent after a certain number of rounds in repeated experiments (Gächter et al. 2008).

## Methods

Participants were Dutch students who were recruited through flyers and posters at the VU University campus in Amsterdam or through an e-mail that was sent to participants in a previous experiment who had indicated that they were willing to participate in future experiments. Flyers, posters, and e-mails contained a hyperlink to a website on which more information was given about the procedure of the experiment. The website contained a further hyperlink to a pre-experimental questionnaire. This questionnaire began with a thorough explication of all stages of the experimental procedure, after which participants signed informed consent. Then, participants filled out a pre-experimental questionnaire.

Online experiments have become more and more common in recent years. The decision to run our experiments online was motivated by two reasons. First, a large sample size is easier to reach online. Second, complete anonymity vis-à-vis other participants, which is an important condition for social dilemma games of this type, is more assured online. Previous research directly comparing online and offline experiments has shown that performing experiments online or in the lab does not significantly alter the results (Amichai-Hamburger 2005; Carpenter et al. 2009; Koopmans and Rebers 2009). Further, Isaac et al. (1994) have shown that increasing the time span of an experiment over several days instead of hours, which is one of the consequences of our online design, did not change participants’ behavior.

Experimenters continued recruiting participants until a total of 180 students had indicated they wanted to participate in the experiment. Then, experimenters formed groups of six randomly chosen participants. Within a week after a participant filled in the pre-experimental questionnaire, he or she would receive an e-mail containing a hyperlink to a website containing the prisoner’s dilemma stage of the experiment. Participants were allowed two working days to fill in this stage. After these 2 days, participants would receive another e-mail with a hyperlink to the second stage of the game, the punishment stage. Participants who did not fill in a specific stage within the allotted time frame were excluded from further participation in the experiment. We set their decision in the PD or IPD to “group account” in cases in which they did not complete this stage, and to “no punishment” if they did not complete the punishment stage. Of course, these default decisions for participants who dropped out do not enter into our results. They were only used to determine the earnings of participants at the end of the game.

To ensure that participants understood the structure of the game, four test questions had to be answered correctly before they could make decisions. After all stages had been completed, decisions made by participants were collected, and participants were paid via internet banking. Participants were paid €4.00 for the pre-experimental questionnaire, provided they participated in the complete experimental game as well. This ensured that participants would at least get a decent base payment for participation independent of their earnings in the experimental game. Payoffs from the experimental game depended on the decisions a participant made, the decisions made by payoff group members, and, in the intergroup competition condition, the decisions made by members of the competing group.

### The Prisoner’s Dilemma Stage

Our version of the Intergroup Prisoner’s Dilemma (IPD) is a variation on that of Bornstein and his colleagues (Bornstein 1992; Bornstein and Ben-Yossef 1994). The main difference is that we used groups of six individuals instead of three in order to decrease the impact one individual had on the public good and thus to increase the similarity between the experiment and real-life collective action dilemmas. Two groups of six participants competed against each other. Each participant received an endowment of €2.00, which he or she could contribute to a group account (cooperation) or put in a private account (defection). Each group had one group account, and each participant had his or her own private account. Money put in the group account increased the portion of a monetary bonus of €43.20 one’s group earned. The portion of the bonus that a group received was divided among all group members independent of whether or not individuals had cooperated. A participant who put his or her money in the private account received both his or her endowment of €2.00 and a share of the group bonus. Table 1 shows payoffs participants received when they put their endowment in the group account or the private account, depending on the number of cooperators in both groups. As can be seen, defectors always received €2.00 more than cooperators in the same group. Further, the table shows that when everyone in group A cooperated and everyone in group B defected, group A received the complete monetary bonus of € 43.20 (€ 7.20 per group member), whereas group B received nothing. Further, if both groups put equal amounts of money in the group accounts, each group received half of the bonus. For intermediate differences, groups received their bonuses accordingly. Regardless of how much money the other group put in the group account, within one’s own group the game was structured as a social dilemma.

**Table 1:** Payoffs for a participant in the intergroup prisoner’s dilemma by chosen account and difference in group accounts

Monetary difference in group accounts (in euros; (group A minus group B)	Payoff in chosen account (in euros)	
	Group account (cooperation)	Private account (defection)
12.00	7.20	
10.00	6.60	8.60
8.00	6.00	8.00
6.00	5.40	7.40
4.00	4.80	6.80
2.00	4.20	6.20
0.00	3.60	5.60
−2.00	3.00	5.00
−4.00	2.40	4.40
−6.00	1.80	3.80
−8.00	1.20	3.20
−10.00	0.60	2.60
−12.00		2.00

### Single-Group Games

In the single-group-with-low-payoff game, six participants play a prisoner’s dilemma. The payoffs of this game are similar to those in the competition game when all group members in the other group put their money in the group account (Table 2).

**Table 2:** Payoffs for a participant in the single-group-low-payoff game by chosen account and amount of money on the group account

Money in group account (in euros)	Payoff in chosen account (in euros)	
	Group account	Private account
12.00	3.60	
10.00	3.00	5.00
8.00	2.40	4.40
6.00	1.80	3.80
4.00	1.20	3.20
2.00	0.60	2.60
0.00		2.00

The single-group-high-payoff game is similar to the single-group-low-payoff game except that the payoffs are similar to the situation in the competition game in which the other group put €0.00 in their group account. For payoffs, see Table 3.

**Table 3:** Payoffs for a participant in the single-group-high-payoff game by chosen account and amount of money on the group account

Money in group account (in euros)	Payoff in chosen account (in euros)	
	Group account	Private account
12.00	7.20	
10.00	6.60	8.60
8.00	6.00	8.00
6.00	5.40	7.40
4.00	4.80	6.80
2.00	4.20	6.20
0.00		5.60

The two PD games and the IPD are structurally similar: in all games, putting the endowment in the private account pays €2.00 for the individual participant, and putting money in the group account pays €0.60 for each group member (including the contributor) from the group bonus. There are two single-group games (not just one) to control for possible effects of the absolute size of the payoffs.

### Punishment Stage

In the punishment stage, participants were allowed to punish group members. Of course, the term “punishment” was not used. Instead, participants were told they had the opportunity to reduce other group members’ payoffs. In line with earlier research (Fehr and Fischbacher 2003; Fehr and Gächter 2002; Henrich et al. 2006; Shinada et al. 2004), the costs of being punished were three times the costs of administering punishment. This level of “punishment effectiveness” has been shown to be sufficient to increase within-group cooperation (Egas and Riedl 2005; Nikiforakis and Normann 2008). Punishment was allowed in increments of €0.10, thereby reducing a group member’s payoff by increments of €0.30. Punishments had to be paid for by the earnings in the PD or IPD. Thus the more money one makes, the more one can punish. Further, a group member could not be punished more than the amount of money he or she earned in the first stage. The punishment round ended with a short post-experimental questionnaire. Subsequently, participants were paid via internet banking.

Of the 180 students who indicated they wanted to participate in the experiment, 147 actually participated. Of those 147 participants, 130 completed all stages of the experiment. We omitted the data of eight participants because they did not follow the rules of the game; either they spent money to punish themselves, they spent more money to punish other group members than was allowed, or, as happened in one case, because the same participant participated twice. Our analysis is therefore based on the decisions of 122 subjects: 40 in the single-group-low-payoff condition, 40 in the single-group-high-payoff condition, and 42 in the competition condition. A chi-square analysis revealed that the dropout level did not differ between single-group and competition conditions (*p* = 0.736).

### Pilot Experiment

To ensure that the facts that our groups were larger and that we conducted the experiment online did not preclude comparison with the study of Bornstein and Ben-Yossef (1994), we first replicated their study without punishment. In other words, we conducted the three conditions of our experiment (single-group-low-payoff, single-group-high-payoff, and intergroup competition) without the punishment stage. After excluding three participants who had not completed the entire experiment, 29, 28, and 30 participants, respectively, remained in the single-group-low-payoff, single-group-high-payoff, and competition conditions. In these three conditions, respectively 38 %, 36 %, and 70 % of the participants contributed their endowment to the group account. Mann-Whitney U-tests indicate no significant difference in cooperation levels between the two single-group conditions (U = 397.00; *p* = 0.864), and that the difference between the intergroup competition and single-group conditions was highly significant (U = 571.500; *p* = 0.003). Participants were thus significantly more likely to contribute their endowment to the group account in the competition condition compared with the single-group conditions. This replicates the two key findings of Bornstein and Ben-Yossef's study. We therefore conclude that group size and the online setting of our experiment do not bias our results.

## Results

In the single-group-low-payoff, single-group-high-payoff, and competition conditions, respectively 40 %, 43 %, and 53 % of the participants contributed their endowment to the group account. A Mann-Whitney U-test showed that in line with Bornstein and Ben-Yossef (1994), and in line with our pilot experiment, there was no significant difference in cooperation levels between the two single-group conditions (U = 1005.500, *p* = 0.791). However, in contrast to these findings on the effects of intergroup competition in situations without punishment, we do not find a significant difference between the cooperation levels in single-group and competition conditions (U = 1977.500, *p* = 0.202).

In the punishment stage, participants in the low-payoff, high-payoff, and competition conditions respectively spent €0.22, €0.24, and €0.45 to punish other group members. Of these amounts, respectively €0.17, €0.17, and €0.39 were used to punish defectors. A linear regression analysis reveals a significant difference between the two single-group and the competition conditions (B = 21.571, *p* = 0.041) Participants on average spent €0.04, €0.07, and €0.07 to punish cooperators. The difference between the two single-group and the competition conditions is not significant (B = 1.405, *p* = 0.762). Cooperators on average spent more money on punishment (€0.39) than defectors (€0.22).

These results are in line with the expectations of hypotheses 1 and 2, but before we accept these hypotheses, we want to put them to a more refined test. As a first step, we check whether the interdependence between the individuals playing a prisoner’s dilemma in one group makes it necessary to use multi- instead of single-level analyses. Such interdependence can occur because all participants in a group received the same information (and different information than members of other groups) about cooperating and defecting behavior of group members in the prisoner’s dilemma stage. A chi-square test revealed that a two-level model, with amount of money spent on punishing defecting group members as dependent variable, indeed had a significantly better fit than a single-level model (*p* = 0.013), which implies that it is necessary to use multi-level regression analyses in which we distinguish two hierarchical levels: the lower level of the individual and the individual’s decisions and the higher level of the group in which the individual played the prisoner’s dilemma. For the analysis of the determinants of punishment of cooperators, preliminary analyses showed that multilevel analysis is not necessary because there was no significant variance at the group level. We therefore use standard linear regression analysis to test hypothesis 2.

### Hypothesis 1

Model I in Table 4 shows that in a multilevel model the effect of competition on the level of punishment of defectors is significant (*p* = 0.074; two-tailed), given that the hypothesis is directional. In model II, we control for some of the variables that are likely to influence the dependent variable. First, the actual number of defectors in the group may affect punishment levels because it reflects the number of punishment opportunities. Second, the amount of money a participant made in the prisoner’s dilemma stage indicates the resources that an individual can employ to punish others. Third, we control for whether or not someone contributed to the group account in the prisoner’s dilemma stage, since our preliminary analyses indicated that cooperators are more likely to punish defectors. The first and third of these variables indeed have a significant positive influence on the dependent variable. In the third model, we add the expectations participants had about the punishment level of their group members. This variable was measured by asking the participants the following question in a post-experimental questionnaire: “How much money do you think the other group members on average spent on punishing other group members?” In the context of public goods the expected contribution levels of others are known to be important predictors for people’s contribution levels (Klandermans 1984, 1997; Koopmans and Rebers 2009; Yamagishi and Kiyonari 2000). Adding this variable to model III indeed shows that the more participants expect others to spend on punishment, the more they themselves spend on the punishment of defectors. The large increases in the explained variances on both levels show the importance of this variable. Controlling for the expected punishment level, the effect of the competition condition becomes highly significant at the 5 % level (*p* = 0.034), which indicates that, at similar expectation levels, people spent more money on the punishment of defectors in conditions with intergroup competition.

**Table 4:** Results of multilevel regression analyses with the amount of money spent on punishing defecting group members as the dependent variable. Reported in the table are (unstandardized) regression coefficients, with significance levels in parentheses. The empty model has both group and individual levels, without independent variables

	Empty model	Model I	Model II	Model III	Model IV
Intercept		16.369 (0.037)	−43.469 (0.196)	−53.778 (0.054)	−47.336 (0.084)
Competition condition		23.806 (0.074)	24.669 (0.054)	22.425 (0.034)	11.440 (0.311)
Number of defectors in group			13.404 (0.016)	10.849 (0.018)	10.348 (0.021)
Earnings in the PD/IPD			0.018 (0.637)	0.020 (0.529)	0.019 (0.529)
Decision: group account			31.139 (0.014)	24.777 (0.016)	22.971 (0.024)
Expectation				0.619 (0.000)	0.491 (0.000)
Expectation*competition condition					0.326 (0.039)
Residual variance (group level)	563.879	467.375	407.487	278.950	262.088
% reduction of variance		17.114	27.735	50.530	53.521
Residual variance (individual level)	2514.314	2495.225	2305.132	1514.160	1466.496
% reduction of variance		0.759	8.320	39.778	41.674
Number of groups	30	30	30	30	30
Number of participants	122	122	122	122	122

Previous research has shown that not only are higher contribution levels to public goods predicted by higher expected contribution levels of group members, but individuals also react more strongly to these levels in conditions in which ingroup biases are salient by giving people information on the cultural similarity between themselves and other group members (Koopmans and Rebers 2009). We suspect that this mechanism may also operate in situations of intergroup competition, which also raises the salience of group boundaries. We therefore introduce in the fourth model an interaction term between the competition condition and expected punishment levels. Indeed, we find that this interaction term is significant, implying that participants react more strongly to expectation levels in the competition condition than in the single-group conditions. The direct effect of the competition condition now becomes insignificant. This implies that punishment behavior of participants in the competition condition is more strongly conditioned by expectations they have about others’ behavior than punishment behavior in the single-group conditions is.

In additional analyses (not reported in the table) we also controlled for sex, age, and whether or not a participant had participated in one of our previous experiments. Introduction of these variables does not affect any of the reported results. We therefore conclude that hypothesis 1 is confirmed: intergroup competition increases the level of punishment directed at defectors. However, the effect is not strong: it only explains a small part of the variance and can be fully explained away by the stronger reaction to expected punishment levels in the competition condition.

### Hypothesis 2

As Table 5 shows, the amount participants spent on punishing cooperating group members did not differ significantly between the competition and single-group conditions. We also find that, though cooperators spent more money on the punishment of defectors, cooperators and defectors spend equal amounts on the punishment of cooperators. When controlling for expected punishment levels, the number of cooperators in a group has a significant influence on the amount spent on punishing them. Again, expected punishment levels strongly influence the actual amount spent on the punishment of cooperators. The interaction term between competition and expected punishment levels remains insignificant. These findings remain similar when controlling for sex, age, and whether or not a participant had participated in one of our previous experiments (not reported). We can thus conclude that hypothesis 2 is confirmed: intergroup competition does not lead to increased punishment of cooperators.

**Table 5:** Results of linear regression analyses with the amount of money spent on punishing cooperating group members as the dependent variable. Reported in the table are (unstandardized) regression coefficients, with significance levels in parentheses

	Model I	Model II	Model III	Model IV
Intercept	5.500 (0.045)	−6.893 (0.386)	−16.080 (0.029)	−15.345 (0.038)
Competition condition	1.405 (0.762)	0.746 (0.873)	0.282 (0.946)	−2.184 (0.664)
Number of cooperators in group		3.449 (0.102)	4.308 (0.023)	4.406 (0.021)
Earnings in the PD/IPD		0.005 (0.719)	0.006 (0.663)	0.006 (0.658)
Decision: group account		−2.332 (0.684)	−4.899 (0.341)	−5.270 (0.308)
Expectation			0.230 (0.000)	0.201 (0.000)
Expectation*competition condition				0.074 (0.383)
R^2^	0.001	0.036	0.240	0.245
Number of participants	122	122	122	122

## Conclusion and Discussion

In this experiment we found support for the two hypotheses that we derived from the direct intergroup competition variant of the cultural group selection perspective. We found confirmation for the expectation that in situations of between-group competition, people spend more resources on the punishment of defecting group members. Also in line with the expectations, we found that between-group competition does not increase the resources people spend on the “perverse” punishment (Cinyabugama et al. 2006) of cooperative group members.

The punishment of defectors can be seen as a form of collective action (Yamagishi 1986) and follows the expected pattern in situations of intergroup conflict (Fehr and Fischbacher 2003). The fact that people are willing to punish cooperating group members at all seems difficult to explain from a group selection perspective, since it decreases group efficiency (Gächter and Herrmann 2009; Herrmann et al. 2008). However, the fact that only the group-beneficial form of punishment, namely that directed toward defectors, increases with intergroup competition, whereas intergroup competition has no effect on the punishment of cooperators, is in line with a group selection perspective.

The finding that defectors are more heavily punished in situations of between-group competition is in line with a result reported by Marlowe et al. (2008), who played experimental economic games in 12 societies and found that members of two of these societies showed a relatively high willingness to punish. The authors suggested that this was due to the recent history of warfare, a form of intergroup competition, in these societies. The scarce field evidence for the effect of intergroup competition on punishment thus seems to be consistent with the theory of cultural group selection.

The prediction that intergroup competition increases within-group punishment is shared by Lahti and Weinstein (2005). They argue that the smaller the chance that the group in which an individual is embedded persists, the more likely it should be that this individual will contribute to the group’s welfare. The same argument has been explicated by Van Veelen and Hopfensitz (2007). In both papers it has been noted that cooperative behavior can evolve because an individual’s chance of survival depends on the group’s chance of survival in cases of intergroup competition. Van Veelen and Hopfensitz argue that this mechanism should be referred to as a form of group selection. Lahti and Weinstein, however, argue that since the group’s persistence is in the individual’s best interest, the mechanism is a form of individual-level selection. We agree that individual-level selection can play a role in the evolution of cooperative behavior if an individual’s cooperative act is crucial for the group’s survival. When groups are larger, however, an individual’s cooperative act is rarely crucial for the group’s survival. Yet, the more individuals cooperate, the higher the chance of the group’s survival in situations of (direct or indirect) between-group competition. We therefore argue that this mechanism can also explain the evolution of collective action, in which an individual’s impact on the public good is always negligible (Olson 1965), and that it should be considered a form of group selection.

In contrast to the punishment of defecting group members, the punishment of cooperating group members cannot be explained by a group-selection perspective: in the long run, the punishment of cooperators decreases the group’s welfare (Gächter and Herrmann 2009; Herrmann et al. 2008). Many possible motivations for perverse punishment have been identified in the literature (for an overview, see Gächter and Herrmann 2009). Research is needed to identify their importance. Revenge for being punished is probably the most commonly suggested motivation (Denant-Boemont et al. 2007; Herrmann et al. 2008; Nikiforakis 2008), but it is not possible in our experiment because we only implemented one punishment round. Our experiment cannot exclude other motivations for perverse punishment, such as concerns for dominance or a preference for higher relative payoffs. The important finding of our experiment, however, is that whatever the motivation for perverse punishment is, it is not influenced by between-group competition.

The findings of our experiment are thus in line with the predictions of a cultural group selection argument. However, this does not imply that they cannot also be interpreted in light of other ultimate explanations. Some authors, for instance, have argued that individual-level selection mechanisms might be able to explain the evolution of cooperative and punishment behavior in small-scale societies, and that the psychological propensities thus evolved continue to structure modern human behavior in situations of anonymous and large-scale human cooperation (e.g., Hagen and Hammerstein 2006; Tooby and Cosmides 1989). When different ultimate explanations predict a similar behavioral pattern, empirical findings such as ours are unable to differentiate between them. Additional research is therefore needed to investigate the plausibility of different ultimate explanations to explain our empirical findings.

Although we found that punishment patterns were in line with the cultural group selection hypothesis, it must be noted that the effect of intergroup competition on the punishment of defectors remains small. First, the effect of intergroup competition on the punishment of defectors is fully caused by the stronger conditionality of punishment on expected punishment levels in the competition condition. Second, expected punishment levels explain a much larger share of the variance in the data than whether or not there is intergroup competition. Apparently, not only are contributions to public goods largely determined by what one expects others to contribute (Klandermans 1984, 1997; Koopmans and Rebers 2009; Yamagishi and Kiyonari 2000), but the group-beneficial act of punishing defectors is also largely determined by how much one expects others to punish. To the best of our knowledge, our experiment is the first to show this effect.

There has been some discussion regarding whether asking participants how much they expect others to contribute really elicits their actual expectations. Some authors have suggested that participants project their own behavior onto others’—the so-called false consensus effect, which in turn explains the correlation between the answers to this question and cooperative behavior of participants (Dawes et al. 1977). Although we agree that part of the “expectations” could be projections, we are convinced that at least part of it represents real expectations. First, the correlations between expectations and contributions differ between specific contexts in which differential projections of own behavior on others are unlikely. For instance, an experiment showed that when the fear of getting the “sucker’s payoff” was made more relevant in a social dilemma, the correlation between expectations and contributions was stronger than in a similar social dilemma in which this fear was made irrelevant (Yamagishi and Sato 1986). This suggests that those who contribute only when they expect others to do so as well try to avoid being the “sucker,” which indicates that expectations influence their own behavior instead of being a mere reflection of it. The differential correlations between situations with and without punishment is another example (Heldt 2005b). Second, experiments in which expectations are not elicited in a questionnaire but with the strategy method show the same conditional cooperation pattern as in our experiment: the more others contribute, the more people are willing to contribute (Fischbacher and Gächter 2010; Fischbacher et al. 2001). Third, manipulating people’s expectations by giving people false information about others’ contributions influences people’s own contributions. The higher others’ presumed contributions, the higher participants’ contributions (Bicchieri and Xiao 2009; Frey and Meier 2004; Heldt 2005a; Martin and Randal 2008). Fourth, general trust in others increases cooperation in real life (Fukuyama 1995; Joireman et al. 1997; Putnam 1993). The above evidence gives us confidence that the expectations we elicited from our subjects to a large extent represent real expectations. In line with many other authors, we therefore argue that many people contribute to collective action depending on how much they expect others to do so (Bogaert et al. 2008; Ellers and Van der Pool 2010; Fischbacher et al. 2001; Gächter 2006; Herrmann and Thöni 2009; Kocher et al. 2008; Koopmans and Rebers 2009; Yamagishi et al. 2008).

Our results show that people react more strongly to expectations in situations of intergroup competition. This finding confirms earlier research on contributions to public goods in single groups, in which group composition varied with respect to culturally transmittable traits (Koopmans and Rebers 2009). In this research, people contributed more to the public good when it benefitted cultural ingroup members, but this could be fully explained by higher expected cooperation levels of other group members, on the one hand, and a stronger reaction toward those expectations, on the other. The results of the current experiment are strikingly similar, as we found that punishment of defectors could be explained by expectations of the inclination of other group members to punish and a stronger reaction to these expectations in the intergroup competition condition. These findings suggest that situations in which group boundaries are made salient—by way of cultural similarity or intergroup competition—lead to higher contribution levels—in the form of contributions to the public good or altruistic punishment of defectors—by way of the mechanism of expectations regarding the behavior of other group members. In other words, salient group boundaries seem to work by triggering conditional participation in collective action. Our findings point to the need for further research on the interplay between group boundary salience, conditional cooperation and conditional punishment.

Our experiment was aimed at investigating the intrinsic reaction of participants to social dilemmas with and without between-group competition and showed that they intuitively respond to situations of between-group competition with an increased level of punishment to defecting group members. This finding cannot be due to strategic considerations, because we chose a two-stage experiment with one contribution round and one punishment round. The consequence of this choice, however, is that our experiment cannot reveal the long-term effect on cooperation levels of the tendency to punish defectors more in situations of between-group competition. Previous research has shown that in the long run, punishment leads to increased cooperation levels, both because of the fear of being punished and because those who have been punished increase contributions (Fehr and Gächter 2000, 2002). Further research should show whether the increased tendency to punish in situations of between-group competition leads to higher and more stable contribution levels compared with repeated single-group games with punishment.
